# The Result of Vitamin C Treatment of Patients with Cancer: Conditions Influencing the Effectiveness

**DOI:** 10.3390/ijms23084380

**Published:** 2022-04-15

**Authors:** János Hunyady

**Affiliations:** Department of Dermatology, Medical Faculty, University of Debrecen, 4032 Debrecen, Hungary; hunyadi@med.unideb.hu or drhunyadij@gmail.com

**Keywords:** cancer, intravenous vitamin C, high-dose vitamin C therapy, clinical trials

## Abstract

Vitamin C (ascorbic acid, AA) is a weak sugar acid structurally related to glucose. All known physiological and biochemical functions of AA are due to its action as an electron donor. Ascorbate readily undergoes pH-dependent autoxidation creating hydrogen peroxide (H_2_O_2_). In vitro evidence suggests that vitamin C functions at low concentrations as an antioxidant while high concentration is pro-oxidant. Thus, both characters of AA might be translated into clinical benefits. In vitro obtained results and murine experiments consequently prove the cytotoxic effect of AA on cancer cells, but current clinical evidence for high-dose intravenous (i.v.) vitamin C’s therapeutic effect is ambiguous. The difference might be caused by the missing knowledge of AA’s actions. In the literature, there are many publications regarding vitamin C and cancer. Review papers of systematic analysis of human interventional and observational studies assessing i.v. AA for cancer patients’ use helps the overview of the extensive literature. Based on the results of four review articles and the Cancer Information Summary of the National Cancer Institute’s results, we analyzed 20 publications related to high-dose intravenous vitamin C therapy (HAAT). The analyzed results indicate that HAAT might be a useful cancer-treating tool in certain circumstances. The AA’s cytotoxic effect is hypoxia-induced factor dependent. It impacts only the anoxic cells, using the Warburg metabolism. It prevents tumor growth. Accordingly, discontinuation of treatment leads to repeated expansion of the tumor. We believe that the clinical use of HAAT in cancer treatment should be reassessed. The accumulation of more study results on HAAT is desperately needed.

## 1. Introduction

The earliest experience of using high-dose vitamin C (intravenous [i.v.] and oral) for cancer treatment was by Cameron, and Campbell, in the 1970s [1]. Later, Cameron and Pauling published the prolongation of survival times in terminal human cancer by supplemental ascorbate in the supportive treatment of cancer [2,3]. In contrast, in the late 1970s and early 1980s, Creagan, Moertel, and O’Fallon, et al. published the failure of high-dose vitamin C therapy (HAAT) to benefit patients with advanced cancer [4,5]. However, these were double-blind studies, while the publications of Cameron, Campbell, and Pauling only matched controlled. Therefore, the publications demonstrating effectiveness have not been accepted for decades. The results were publicly debated with considerable acrimony between Pauling and Moertel, with accusations of misconduct and scientific incompetence on both sides.

Later it was confirmed that oral and parenteral administration of ascorbate is not comparable. The serum level of AA is significantly lower after a high oral AA dose than after a high dose of i.v. AA [6]. This difference explains the different results observed by Creagan and Cameron, as Creagan/Moertel used oral [4,5], while Cameron/Pauling oral + parenteral AA [1,2,3]. Although Cameron’s treatment was mainly oral therapy, they used i.v. AA as an initiation to the use of oral AA. The i.v. AA treatments lasted only up to 10 days, and only 2/3 of subjects received it [1].

The study by Levine M et al. [7] showed that when vitamin C is ingested by mouth, plasma and tissue concentrations are tightly controlled by at least three mechanisms in healthy humans: absorption, tissue accumulation, and renal reabsorption. With ingested amounts found in foods, vitamin C plasma concentrations usually do not exceed 100 μmol/L. Even with supplementation approaching maximally tolerated doses, ascorbate plasma concentrations are always <250 μmol/L and frequently <150 μmol/L. By contrast, when AA is i.v. injected, tight control is bypassed until excess ascorbate is eliminated by glomerular filtration and renal excretion. With i.v. infusion, pharmacologic ascorbate concentrations of 25–30 mmol/L are safely achieved. Pharmacologic AA can act as a pro-drug for H_2_O_2_ formation, leading to an extracellular fluid at levels as high as 200 μmol/L. In addition, pharmacologic ascorbate can elicit cytotoxicity toward cancer cells and slow tumor growth in experimental murine models. See review by Levine et al. [7]. 

Pharmacologic ascorbate sensitizes cancer cells to damage by increasing intracellular L-ascorbate engagement through sodium-dependent vitamin C transporter 2 (SVCT-2) by acting as a pro-drug [7]. HAAT is an aggressive adjunctive cancer treatment, widely used in naturopathic and integrative oncology settings, although it is not accepted as an effective drug for cancer patients by health authorities (FDA, EMA).

Until today, many publications are available regarding vitamin C treatment of malignant tumors. However, in vitro and animal experiments have proved AA’s pharmacological cytotoxic effect on cancer cells, while the human clinical observations and studies show contradicting data. Thus, the Pauling–Moertel debate continues to this day.

This article summarizes the mechanism of the effects of sugar withdrawal (fasting, ketogenic diet, blocking of glucose transporters) and i.v. AA treatment on cancers. It reviews the hypothetical causes of the difference between in vitro and animal studies compared to human outcomes. Finally, it points out that confirming the clinical effect is possible by carrying out further clinical trials and summarizing the conditions considered when planning a trial.

## 2. The Vitamin C Treatment’s Theoretical Framework

### The Biochemical Motor for Energy Transformation

Previously, we described a hypothetical structure, the structure for energy transformation (SET), which might be responsible for the proper energy transformation, leading to the continuous membrane potential, production of H^+,^ and ATP in living cells [8]. We suppose that the SET of aerobic glycolysis (SET-AG) is responsible for managing aerobic glycolysis, the SET of oxidative phosphorylation (SET-OP) for the process of oxidative phosphorylation. The ADP producing unit (ADP-PU) is the basic structure of both SETs. Six D-glucose and two L-AA are essential for the proper function of the ADP-PU. We suppose that the ribose of adenine originates from glucose. AA starts the transformation by producing O^2e−^ through its effect on Fe-S clusters. The absence of glucose results in the accumulation of O^2e−^, which might cause cell death.

The ratio change (lower glucose content) might result in excess H_2_O_2_ production, leading to cell death since unused O^e2−^ will remain in the cell in the absence of glucose [8] (see Supplementary Materials).

## 3. The Dual Energetics of Eucaryotic Cells

### Symbiosis of One Ancient Cell with a New Cell Resulted in Eukaryote

The defense of the first living cells against free radicals was low, as O_2_ was not present in the environment. Thus, the emergence of O_2_ produced by cyanobacteria resulted in an environmental disaster, and most living cells died. Simultaneously, new cells armed with an effective defense system against free radicals were created. In addition, these cells could produce significantly more energy using O_2_ than ancient cells [9]. Lynn Margulis believed that the eukaryote cells’ ancestors avoided being destroyed by oxygen by entering a symbiotic relationship with the new aerobic bacteria, known today as the mitochondrion. Eukaryotic cells are equipped with the SET-AG and the SET-OP; thus, they can live in an anoxic and oxygenized environment [10]. Mitochondria provide the cell with a significantly better energy supply and free radical protection.

## 4. Development of Cancer

Cancer is a group of diseases involving abnormal cell growth to invade or spread to other parts of the body. More than 100 types of cancer are usually named for the organs or tissues where the tumors form. For a normal cell to transform into a malignant cell, the genes that regulate cell growth and differentiation must be altered [11]. The affected genes are oncogenes or tumor suppressor genes [12].

### 4.1. Protective Options against Malignant Tumors

Living organisms continuously defend against harmful environmental factors. There are specialized systems (such as apoptosis) for controlling both the invaders and the abnormally functioning cells. In animals (and in humans), immune cells can usually recognize and destroy abnormal cells. Unfortunately, there are many situations when malignant cells escape from the control of the defense and multiply without control leading to death [13].

### 4.2. The Chain Reaction of Cancer-Development

Clonally evolution leads to intra-tumor heterogeneity. The genetic properties and the states of the tumor cell metabolism (oxidative phosphorylation or aerobic glycolysis) are different. This heterogeneity complicates designing effective treatment strategies. 

**Figure 1:**
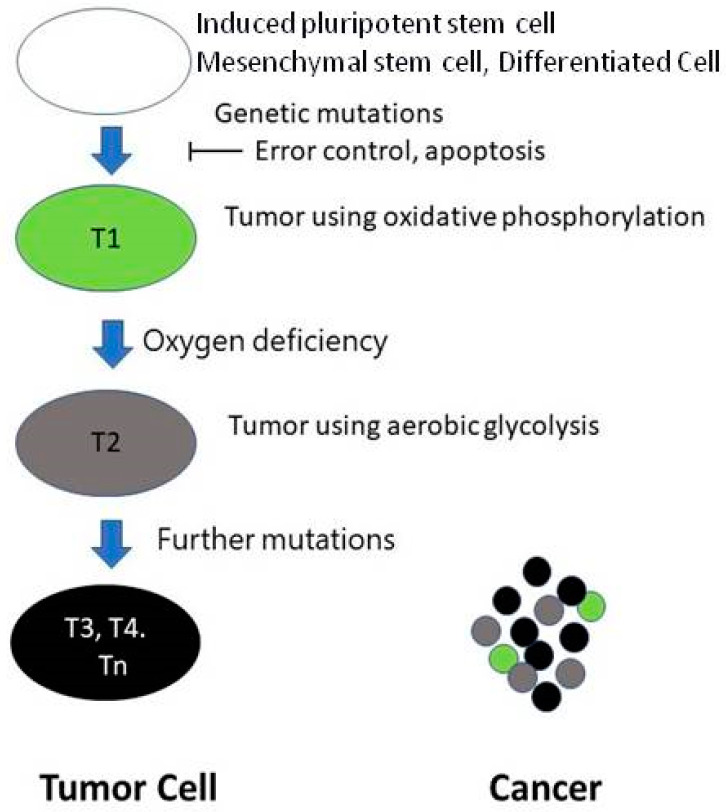
Evolution of malignant tumor cells.

In developing a malignant tumor, the first step is the appearance of a mutant cell. The second problem is that the error control mechanism is altered. Thus, the mutant cell is not eliminated [14]. The multiplying mutant cells are the progenitor cells of the tumor (T1), using oxidative phosphorylation. The distance between the T1 containing growing tumor cells and the supplying blood vessel is continually increasing. The growing new tumor has no vessels. Thus, at a certain point, the partial tissue pressure of the O_2_ (pO_2_) level will not be enough for the proper mitochondrial function [15]. At the same time, hypoxia-induced factor-1 alpha (HIF-1α) will not be hydrolyzed, resulting in the further development of the tumor; cells able to live in an anoxic environment (tumor stem cells) will be created (T2). The HIF system will further develop the tumor, creating tumor cells with new behaviors (T3, T4, Tn). Between these cells, competition exists. Cancer is mainly formed by highly malignant cells, but other cells, including T1 with oxidative phosphorylation, are also present in the tumor (Figure 1).

In the presence of oxygen in the cells of differentiated tissues, sugar becomes pyruvate, and then CO_2_ and energy by oxidative phosphorylation. Conversely, in the absence of O_2_, aerobic glycolysis is initiated instead of mitochondrial oxidative phosphorylation, leading to lactate formation and heterogeny of tumor cells [16].

Because aerobic glycolysis produces significantly less energy, cells can only be viable by using more sugar. Tumor cells use 200 times more glucose than healthy ones [17].

Malignant tumor cells perform glycolysis at a ten times faster rate than their healthy tissue counterparts [18]. While rapidly growing tumor cells do not have adequate vessels during their genesis, the limited capillary support often results in hypoxia within the tumor. In addition, some tumor cells overexpress specific glycolytic enzymes, resulting in higher glycolysis rates, referred to as the Warburg effect [19].

The most common cellular metabolism changes involve intracellular glucose utilization and regulation loss between glycolytic metabolism and respiration [20]. Thus, tumor cells adapted to the hypoxic environment by the HIF-1α have unique energy production, realized by the low-efficiency aerobic glycolysis. 

AA forms dehydroascorbic acid (DHA) after oxidation. The level of vitamin C in cells may be related to vitamin C transporter expression and its polymorphism in cells. In normal human cells, two different transporter systems are responsible for the acquisition of vitamin C: glucose transporters (GLUTs)—GLUT1, GLUT3, and GLUT4—transfer the DHA and SVCTs transport the AA. The variations in vitamin C transporter genes may regulate the active transport of vitamin C and, therefore, impact cancer risk [21].

Several studies indicate the critical role of vitamin C transport by GLUTs in cancer cells. Pena et al. suggested that the vast majority of vitamin C transferred from the extracellular space into cancer cells assumes the form of DHA [22]. Several cell culture studies suggest that DHA transport is an alternate or even principal pathway of vitamin C accumulation [23]. DHA is rapidly reduced into its AA form following diffusion with the simultaneous oxidation of glutathione and NADPH [24]. 

Cho et al. showed that the cellular response to AA treatment was dependent on SVCT2 expression. Cancer cells with low SVCT2 expression levels exhibited anti-cancer effects at high doses of ascorbic acid and a proliferative impact at low doses of this compound. In contrast, cancer cells with high SVCT2 expression exerted anti-cancer outcomes at all ascorbic acid concentrations [25].

## 5. HIF-1α the Conductor of the Extended Malignancy

### A System for Monitoring Oxygenation

Hypoxic stress is a companion to many diseases and its resolution is an essential physiological process. Our body has a protective function that delays the damage caused by hypoxia and ensures the elimination of the hypoxia-damaged tissue. This defense system triggers regeneration, resulting in the survival of cells in the hypoxic zone surrounding the dead area. The key to the system is the HIF-1α molecule. HIF helps develop tumor stem cells (T2) and extend malignancy. Accordingly, the HIF system is the conductor for educating tumor cells on the way to death.

Due to hypoxia or AA deficiency, the protein HIF-1α is activated, and hundreds of stress-related genes will change expression leading to their up- or down-regulation. In addition, it includes genes regulating apoptosis, and, due to HIF-1α, the sensitivity of cells to apoptosis induction will be decreased. In other words, HIF-1α protects cells from the apoptosis caused by hypoxia. Thus, the HIF-1α level in the cells increases exponentially due to inadequate nutritional supply, viral infection, radiation, or other apoptotic signals, and if the oxygen level is less than 6% (partial pressure O_2_ < 40 mmHg) [26,27,28,29]. 

Hypoxia can develop in the body for a variety of causes. In this case, HIF-1α will not be destroyed due to a lack of oxygen. In the nucleus, HIF-1α, together with the HIF-1β unit, produces the HIF-1 protein, which changes the cellular physiological parameters by influencing many genes’ regulation. Angiogenesis, remodeling, cell proliferation, migration, cancer development, cell growth, apoptosis, metabolism, xenobiotic transporter, hematopoiesis, melanogenesis, oncogenes, re-epithelialization, and melanogenesis are the crucial target genes. In addition, several other molecules (such as HIF-2) are involved in complex HIF regulation [15]. 

The physiological system induced by HIF molecules plays an essential role in embryonic development, wound healing, and the course of several diseases (such as infarction, stroke, vasculitis, etc.). Unfortunately, however, this system plays a crucial role in increasing the aggressivity of many malignancies. 

The growing tumor cell is free of control. It gradually moves away from the supplying vessel. When this distance exceeds 70–100 nanometers, the cells’ oxygen supply becomes insufficient [15]. At that point, the failure to hydrolyze HIF-1α will trigger malignant cells for higher malignancy. Thus, the HIF system initiates a regenerative physiological process that results in the restoration of normoxia by vascularization. At the same time, a new type of tumor cell with a more aggressive phenotype adapted to the lack of oxygen will be developed. 

Hypoxia-induced, HIF-mediated neovascularization ensures the tumor’s steady growth. Tumor cells of malignant cancers usually are highly heterogeneous. They can be classified into “normoxic/oxidative” or “hypoxic/aerobe glycolytic” cells. The treatment, targeting the HIF system, controls only selected cells of the tumor (cells using aerobic glycolysis) and prevents the growth but does not destroy cancer.

## 6. Treatment of Tumors by Influencing Glucose Metabolism

In most cancer cells, especially the most aggressive phenotypes, there is a substantial uncoupling of glycolysis from oxidative phosphorylation with the consequent production of high lactate levels (Warburg effect). This metabolic modification gives the tumor an evolutionary advantage, adapting to the more-or-less transient hypoxic conditions occurring during the disease’s progress. Furthermore, the remarkably higher glucose uptake also shows this metabolic preference by cancer cells through transmembrane glucose transporters, compensating for the higher energy demand of rapidly growing cells. Due to this feature, any enzyme or transporter promoting the glycolytic flux may be considered a potential target to block tumor progression [30,31,32].

Recently, human cancer cells were shown to be more susceptible to glucose deprivation-induced cytotoxicity and oxidative stress relative to non-transformed human cell types. These results indicated that some biochemical processes provided a mechanistic link between glucose metabolism and the expression of phenotypic characteristics associated with malignancy [30,31,32]. 

The most direct way to target exaggerated aerobic glycolysis in tumors is to reduce glucose availability to cancer cells. It can be achieved through either dietary or pharmacological interventions. The pharmacologic influence might be realized either by controlling glucose uptake of the tumor cell by inhibiting glucose transporters or by inhibiting glycolytic enzymes [33,34].

### 6.1. Short-Term Fasting

Short-term fasting (STF) 48 to 72 h before chemotherapy appears to be more effective than intermittent fasting. Preliminary data show that STF is safe but challenging in cancer patients receiving chemotherapy. Ongoing clinical trials need to unravel if STF can also diminish the toxicity and increase chemotherapeutic regimens’ efficacy in daily practice [33].

### 6.2. Ketogenic Diet

The ketogenic diet consists of high fat, moderate to low protein, and extremely low carbohydrates. The goal is to develop ketosis in the body. It occurs when the body is forced to use fat for energy without glucose [34]. 

### 6.3. Clinical Trials of Fasting

There is currently no concrete evidence that any of these fasting diets will benefit cancer patients. Clinical trials with more patients are still needed before fasting could be used in standard practice [35]. 

### 6.4. Glucose Transporters

The glucose entrance inside the cell occurs by facilitated diffusion and mainly depends on GLUTs. There are three different classes of GLUTs with tissue-specific distribution and distinct affinity for glucose and other carbohydrates. Class 1 comprises four members, GLUT1–LUT4, whose preferential substrate is glucose, while the other two classes, class 2 (GLUT5) and 3 (GLUT6, 8, 10), are more selective for other sugars [36]. 

GLUTs are expressed 10–12-fold higher in cancer cells than in healthy tissues, especially in highly proliferative and malignant tumors, contributing to the high glycolytic flux observed in this kind of tissue [37]. GLUT1 and GLUT3, whose expression is regulated by HIF-1α, can be considered the main over-expressed isoforms in a wide range of human cancers [38]. Moreover, they are correlated with poor prognosis and the radioresistance of several types of human tumors. Hence, the activation of their expression can be considered a typical feature of the malignant phenotype. GLUT has a fundamental role in tumor cells of aerobic glycolysis. Thus, GLUT inhibition may represent an appealing way of attacking cancer by blocking its direct nutrient uptake, thus reducing the glycolytic flux and causing cell death by starvation and H_2_O_2_.

Antibodies [39], antisense nucleic acids [40,41,42], and synthetic small organic molecules showing GLUT-inhibitory properties, either alone or in combination with chemotherapeutic drugs, have been reported [43].

Numerous observations suggest that starvation, ketogenic diet, and glucose transporter’s inhibition have antitumor activity, but clinical studies to support this are lacking.

## 7. In Vitro and Animal Studies of Pharmacologic Ascorbic Acid

Several studies have demonstrated that AA’s in vitro cytotoxic effect on various cancer cells is mediated through a chemical reaction that generates hydrogen peroxide [7,44,45,46]. Furthermore, cell death from H_2_O_2_ added to cells was identical to that found when H_2_O_2_ was generated by ascorbate treatment [47]. In mice bearing glioblastoma xenografts, a single pharmacologic dose of ascorbate produced sustained ascorbate radical and hydrogen peroxide formation selectively within the interstitial fluids of tumors but not in the blood, indicating H_2_O_2_-mediated cytotoxicity [48]. 

### 7.1. Sodium-Dependent Vitamin C Transporter-2 Sensitizes Cancer Cells to Damage by Increasing Intracellular L-Ascorbate Concentration 

Hongwei et al. [49] demonstrated that L-ascorbate has a selective killing effect influenced by SVCT-2 in human hepatocellular cancer cells [49]. Furthermore, SVCT-2 expression was absent or weak in healthy tissues but strongly detected in tumor samples obtained from breast cancer patients [17].

### 7.2. The Proposed Way of Action by Parenteral AA on Sensitive Cancer Cells 

#### Pharmacologic AA and HIF1α 

The in vitro cytotoxic effect of ascorbic acid’s pharmacological concentration on cancer cells is mediated through a chemical reaction that generates H_2_O_2_ [50]. In addition, ascorbic acid induces reactive oxygen species and impaired mitochondrial membrane potential [47]. 

Vitamin C enters the cell through SVCT-2 [49] and changes the intracellular glucose/AA ratio. The high intracellular AA concentration in the tumor cell results in the proteolysis of the HIF-1α, which will lead to the cessation of the HIF-1α induced low apoptotic sensitivity [51].

The degradation process of HIF-1α is O_2_ and vitamin C dependent. HIF-1α regulates essential genes, including angiogenic/angiostatic-, oxidative stress-, and apoptosis-related genes. It helps neovascularization and suppresses the sensitivity of cells for apoptosis [28]. Pharmacologic concentrations of ascorbic acid cause diverse influences on different angiogenic chemokine expressions [52,53]. For example, the angiostatic gene, CXCL10, was downregulated by the high pharmacologic level of AA in hepatocellular carcinoma cell lines [47]. In addition, AA (1–3 mM) decreases colon cancer cell proliferation and induces apoptosis and necrosis accompanied by downregulation of specificity protein (Sp), including vascular endothelial growth factor (VEGF) and its receptors VEGFR-1 and VEGFR-2 [54]. 

### 7.3. Glutathione and Catalase Result Resistance of Cancer Cells against AA Mediated Cytotoxicity 

Hardaway et al. [55] showed that H_2_O_2_-produced cell death was partly mediated by losing total glutathione levels in the cells. Glutathione reduced cytotoxicity by 10–95% by attenuating AA-induced H_2_O_2_ production. Co-treatment with glutathione inhibits the cytotoxic responses [54]. One study analyzed the impact of catalase on cancer cells’ resistance to ascorbic acid-mediated oxidative stress. The tested human cancer cell lines demonstrated apparent differences in their opposition to AA-mediated oxidative cell stress [56]. 

### 7.4. Ascorbate Sensitivity of Different Cells Is Diverse 

According to in vitro and in vivo data, different cells show a diverse reaction to ascorbate treatment. Tumor cells are usually sensitive to the pharmacological dose of AA, while healthy cells are not. While androgen-independent human prostate cancer cells are sensitive to ascorbate treatment, the ascorbate-insensitive human prostate cancer cell line LaPC4 is hormonally dependent [50]. Predictably, the hormone dependence of other tumor cells might influence their sensitivity to AA’s pharmacological dose. 

### 7.5. Synergistic Interactions of Pharmacologic AA, Cytostatic Drugs, Radio, and Photodynamic Therapy 

Ascorbate treatment specifically enhances the cytostatic potency of certain chemotherapeutics such as docetaxel and 5-FU, gemcitabine [57], epigallocatechin-3-gallate [58], and As_2_O_3_ [59]. One study suggested that high-dose ascorbate increases the radiosensitivity of glioblastoma multiforme cells, resulting in more cell death than from radiation alone [60]. An internally related, complementary, and strengthened tumor treatment is established by combining photodynamic therapy (PDT) and ascorbate as a low-toxicity and effective method. PDT induces metal ion release, and ascorbate reacts with the metal ions producing subsequent ROS [61]. 

In vitro-obtained results and murine experiments consequently prove the cancer cell’s cytotoxic effect of AA, but current clinical evidence for high-dose i.v. vitamin C’s therapeutic effect is ambiguous [7,44,45,46,47,48,62,63,64,65].

### 7.6. Presumed Causes of Differences between In Vitro and Murine Experimental Results Compared to Human Findings

In vitro and animal studies were conducted under standard conditions. Mice and most animals are invalid for AA trials as they can produce AA. Most vitamin C trials in murine models are performed with xenografts and not spontaneous tumors. Unfortunately, there is a lack of standardization of treatment in cancer patients. The supposed determining factors between oral and intravenous administration is neglected. The dose of vitamin C used during therapy varies from study to study. Diabetes and sugar consumption might reduce the effect of vitamin C. We assume that in the case of AA dissolved in a 5% dextrose infusion [66,67], the dextrose might reduce the cytotoxic effect of vitamin C. The defining properties of tumors are not considered. Although the tumor comprises cells with different properties, they contain cells using oxidative phosphorylation and cells characterized by Warburg metabolism. Hormone dependency on cancer might influence the efficacy of the HAAT. Patients with advanced cancer often have abnormal laboratory values (e.g., anemia, lower Se Fe, Se transferrin) that may affect the ascorbate effectiveness.

## 8. Clinical Results Regarding Cancer’s Vitamin C Treatment

In the literature, there are many publications concerning vitamin C and cancer. A quick PubMed search of “vitamin C and cancer” yielded 55,936 results and “ascorbic acid cancer therapy” 30,032 results (07.2021). Review papers of systematic analysis regarding human interventional and observational studies assessing i.v. AA for cancer patients help in the overview of the extensive literature.

Based on the results of four review articles [62,63,64,65] and the Cancer Information Summary of the National Cancer Institute’s results–Health Professional Version [68], we analyzed 20 publications related to HAAT.

We researched the literature on the following fields:

- Effectiveness of HAAT compared to placebo or no treatment in susceptible populations,
- toxicity and side effects of vitamin C,
- Examination of factors suspected of influencing HAAT efficacy such as
  ○ hormone dependency of the tumor,
  ○ the liquid’s composition (physiological NaCl, 5% dextrose, or Ringer’s solution) used in the infusion preparation,
- duration of treatment.

In the first source review, 897 records, 127 full-text articles, and 39 reports were included [62]. The second source review used 690 initial screen records, 61 full-text articles, and 34 final reports [63]. The third source included single-arm and randomized phase I/II trials. They used 517 initial screen records and 34 full-text articles, and 23 final reports [64]. The fourth source used 920 initial screen records and 38 full-text articles, including a19 final reports [65]. Finally, the fifth source contains 18 reports regarding HAAT [68].

In the four review papers, 8, 10, 12, and 19, HAAT publications are available, while the doctor’s National Cancer Institute’s information mentions 18 publications.

The five sources concluded: there is limited/no high-quality clinical evidence on the effectiveness of HAAT. Overall, vitamin C is safe in nearly all patient populations, alone and in combination with chemotherapies. The i.v. AA may improve patients’ quality of life and symptom severity with cancer. Well-designed, controlled studies of HAAT are needed [62,63,64,65].

It is not easy to assess the review papers’ data, as they have been calculated and processed differently. Cameron used i.v. AA only as an initiation to oral vitamin C [1,2,69]. Our main objective was to analyze the data related to HAAT. We collected those publications where AA was used intravenously in high (>2.5 g/day) concentrations.

Our aggregated and supplemented data contain 20 publications regarding HAAT (Table 1).

Summarizing the results of four review articles and the Cancer Information Summary of the National Cancer Institute’s results, we have concluded that HAAT is safe; no toxicity has been observed.

In eleven publications, remarkable clinical results were detected in more patients, while the clinical results were uncertain in two. Cancer regression was not detected in seven publications. Two of them used dextrose to infusion AA, and three of them treated prostate cancer patients.

These results indicate that HAAT might be an effective cancer treatment procedure under certain circumstances. However, we assume that well-signaled conditions influence the HAAT’s effectiveness.

## 9. Supposed Factors Influencing the Haat’s Efficacy

### 9.1. Glucose Dependency

It is known that pharmacologic AA can induce some cancer cell death in vitro and inhibit several types of tumor growth in animal models through the production of H_2_O_2_. It is also known that glucose deprivation as well as i.v. AA might result in benefits for cancer patients. Limited case reports indicate that a ketogenic diet combined with i.v. AA improves the effectiveness of HAAT [78]. Based on these results, it is predictable that the patient’s serum glucose level might influence the effectiveness of i.v. AA therapy. Consumption of carbohydrate-containing food or drink when the serum level of AA is in the toxic range (300 min after finishing the infusion) might prevent the realization of the poisonous effect. Unfortunately, most publications do not contain data relating to the nature of carrier infusion (NaCl, Ringer, Ringer’s lactate, or 5% dextrose). We assume that dextrose might terminate the cytotoxic effect of vitamin C if AA is dissolved in a 5% dextrose infusion [66,67].

### 9.2. Hormone Dependency

All tested androgen-independent cells were sensitive to ascorbate treatment. The ascorbate-insensitive prostate cancer cell line LaPC4 is hormonally dependent. They concluded that high-dose ascorbate could be a novel treatment option for hormone-refractory prostate cancer [50].

We do not know whether an originally hormone-dependent cancer cell can further develop and lose the hormone dependency. The AA sensitivity nature of such a cell is also not known. The negative results regarding prostate cancer patients [52,80] might be related to this.

Hormone dependency of tumors is usually not detailed in the publications.

### 9.3. Duration of Treatment

The AA’s cytotoxic effect is HIF dependent. It impacts only the anoxic cells, using Warburg metabolism, preventing tumor growth. Accordingly, discontinuation of treatment leads to repeated tumor expansion, as previously published [2]. In breast cancer patients receiving VEGF inhibitor treatment, similar progression may be observed if treatment is discontinued.

## 10. Conclusions

In vitro and murine experiments demonstrate AA’s pharmacological doses’ efficacy, but the results of clinical trials are contradictory. HAAT is considered safe; no toxicity has been observed. However, only half of the publications report on clinical efficacy.

### 10.1. The Glucose Deprivation and Vitamin C Therapy’s Way of Action

During energy transfer in cells, O^2e−^ is continuously produced in the presence of glucose and AA. During energy transformation, ribose will be cut from the glucose molecule, forming adenosine. If glucose is not available, the O^2e−^ produced by Fe-S clusters destroys the tumor cell. The situation might be formed by a glucose deficit or a high intracellular concentration of AA [8].

The cell’s ability to protect against free radicals in the presence of O_2_ is significantly higher than that in a low-oxygenated environment due to the intensive mitochondrial defense against ROS action. However, the normoxic tumor cells survive. Therefore, i.v. AA therapy and glucose deprivation should be long lasting and used in combination with conventional anticancer treatments.

HAAT might be an effective cancer treatment procedure under certain circumstances. We assume the HAAT’s effectiveness. The tumor’s nature and hormone dependency, diabetes mellitus, and the liquid’s composition (physiological NaCl, 5% dextrose, or Ringer’s solution) used in the infusion preparation are determining factors.

### 10.2. Suggestion of Well Designed, Controlled Studies of HAAT

We believe that the following factors must be considered when planning HAAT

(a) Conduct AA trials on persons with hormone-independent cancers or stratify randomization on hormone dependence so that effects can be analyzed separately.
(b) Use Ringer’s solution or physiologic NaCl to prepare the infusion, not dextrose.
(c) Record sugar consumption and other dietary factors.
(d) Record the serum glucose level of the patient immediately after finishing the infusion and again at 4 h after finishing the infusion.
(e) Report all these factors and frequency and total duration of treatment in all reports.

## Figures and Tables

**Table 1:** Results of pharmacologic vitamin C therapy.

Study	Participants	Result
Cameron, 1974 [1]	Advanced-stage cancer patients	++
Cameron, 1976 [2]	Incurable cancer patients	++
Cameron, 1991 [69]	Terminal cancer patients	++
Murata, 1982 [70]	Terminal cancer patients	++
Ma, 2014 [71]	Newly diagnosed stage III ovarian cancer after debulking	++
Hoffer, 2015 [72]	Advanced-stage cancer patients	++
Gunes-Bayir, 2015 [73]	Bone metastases from various types of cancer	++
Zhao, 2018 [74]	Newly diagnosed elderly with acute myeloid leukemia	++
Wang, 2019 [75]	Metastatic colorectal cancer or gastric cancer	++
Monti, 2012 [76]	Metastatic stage IV pancreatic cancer	++
Polireddy, 2017 [77]	Locally advanced or metastatic prostate cancer	++
Raymond, 2016 [78]	Wide variation in the severity and type of cancer	+
Schoenfeld, 2017 [79]	Glioblastoma, non-small cell lung carcinoma	+
Welsh, 2013 [66]	Stage IV pancreatic adenocarcinoma.	0
Bazzan, 2018 [67]	All types of cancer in different settings	0
Mikirova, 2012 [52]	Various types of cancer, primarily metastatic prostate	0
Nielsen, 2017 [80]	Chemotherapy-naive metastatic castration-resistant prostate cancer	0
Hoffer, 2008 [81]	Advanced cancer or hematologic malignancy	0
Riordan, 2005 [82]	Late-stage terminal cancer, mostly colorectal	0
Stephenson, 2013 [83]	Advanced solid tumors	0

++: remarkable clinical regression in more patients; +: the clinical effect is uncertain; 0: no clinical result.

## Data Availability

Not applicable.

## Supplementary Materials

The following supporting information can be downloaded at: https://www.mdpi.com/article/10.3390/ijms23084380/s1. References [8,46,84,85,86,87,88,89,90,91,92,93,94,95,96,97,98,99] are cited in the Supplementary Materials.

## References

[1] Cameron E., Campbell A. (1974). The orthomolecular treatment of cancer II. Clinical trial of high-dose ascorbic acid supplements in advanced human cancer. Chem. Biol. Interact..

[2] Cameron E., Pauling L. (1976). Supplemental ascorbate in the supportive treatment of cancer: Prolongation of survival times in terminal human cancer. Proc. Natl. Acad. Sci. USA.

[3] Cameron E., Pauling L. (1978). Supplemental ascorbate in the supportive treatment of cancer: Reevaluation of prolongation of survival times in terminal human cancer. Proc. Natl. Acad. Sci. USA.

[4] Creagan E.T., Moertel C.G., O’Fallon J.R., Schutt A.J., O’Connell M.J., Rubin J., Frytak S. (1979). Failure of high-dose Vitamin C (ascorbic acid) therapy to benefit patients with advanced cancer. A controlled trial. N. Engl. J. Med..

[5] Moertel C.G., Fleming T.R., Creagan E.T., Rubin J., O’Connell M.J., Ames M.M. (1985). High-dose Vitamin C versus placebo in the treatment of patients with advanced cancer who have had no prior chemotherapy. A randomized, double-blind comparison. N. Engl. J. Med..

[6] Verrax J., Calderon P. (2009). Pharmacologic concentrations of ascorbate are achieved by parenteral administration and exhibit antitumoral effects. Free Radic. Biol. Med..

[7] Levine M., Padayatty S., Espey M. (2011). Vitamin C. A concentration-function approach yields pharmacology and therapeutic discoveries. Adv. Nutr..

[8] Hunyady J. (2021). The role of Vitamin C in the energy supply of cells Hypothetical structure for energy transformation 2021. JSRR.

[9] Cooper G.M. (2000). The Cell: A Molecular Approach.

[10] Lynn M. (1970). Origin of Eukaryotic Cells: Evidence and Research Implications for a Theory of the Origin and Evolution of Microbial, Plant, and Animal Cells on the Precambrian Earth.

[11] Croce C. (2008). Oncogenes and cancer. N. Engl. J. Med..

[12] Pettini F., Visibelli A., Cicaloni V., Iovinelli D., Spig O. (2021). Multi-omics model applied to cancer genetics. Int. J. Mol. Sci..

[13] Tellez-Gabriel M., Ory B., Lamoureux F., Heymann M.-F., Heymann D. (2016). Tumour heterogeneity: The key advantages of single-cell analysis. Int. J. Mol. Sci..

[14] Afify S.M., Seno M. (2019). Conversion of stem cells to cancer stem cells: Undercurrent of cancer initiation. Cancers.

[15] Padhani A.R., Krohn K.A., Lewis J.S., Alber M. (2007). Imaging oxygenation of human tumours. Eur. Radiol..

[16] Schwartz L., Seyfried T., Alfarouk K., Da-Veiga-Moreira J., Fais S. (2017). Out of warburg effect: An effective cancer treatment targeting the tumor specific metabolism and dysregulated pH. Semin. Cancer Biol..

[17] Hong S., Lee S., Moon J., Hwang J., Kim D., Ko E., Kim H., Cho I., Kang J., Kim D. (2013). SVCT-2 in breast cancer acts as an indicator for L-ascorbate treatment. Oncogene.

[18] Alfarouk K., Shayoub M., Muddathir A., Elhassan G., Bashir A. (2011). Evolution of tumor metabolism might reflect carcinogenesis as a reverse evolution process (dismantling of multicellularity). Cancers.

[19] Vander-Heiden M., Cantley L., Thompson C. (2009). Understanding the warburg effect: The metabolic requirements of cell proliferation. Science.

[20] DeBerardinis R.J., Chandel N.S. (2016). Fundamentals of cancer metabolism. Sci. Adv..

[21] Muñoz-Montesino C., Peña E., Roa F.J., Sotomayor K., Escobar E., Rivas C.I. (2021). Transport of Vitamin C in cancer. Antioxid. Redox Signal..

[22] Peña E., Roa F.J., Inostroza E., Sotomayor K., González M., Gutierrez-Castro F.A., Maurin M., Sweet K., Labrousse C., Gatica M. (2019). Increased expression of mitochondrial sodium-coupled ascorbic acid transporter-2 (mitSVCT2) as a central feature in breast cancer. Free Radic. Biol. Med..

[23] Agus D.B., Gambhir S.S., Pardridge W.M., Spielholz C., Baselga J., Vera J.C., Golde D.W. (1997). Vitamin C Crosses the Blood-Brain Barrier in the Oxidized Form through the Glucose Transporters. J. Clin. Investig..

[24] Linster C.L., Schaftingen E.V. (2007). Vitamin C. FEBS J..

[25] Cho S., Chae J.S., Shin H., Shin Y., Song H., Kim Y., Yoo B.C., Roh K., Cho S., Kil E.-J. (2018). Hormetic dose response to l-ascorbic acid as an anti-cancer drug in colorectal cancer cell lines according to SVCT-2 expression. Sci. Rep..

[26] Huang L., Gu J., Schau M., Bunn H. (1998). Regulation of hypoxia-C 1alpha is mediated by an O_2_-dependent degradation domain via the ubiquitin-proteasome pathway. Proc. Natl. Acad. Sci. USA.

[27] Knowles H., Raval R.R., Harris A.L., Ratcliffe P.J. (2003). Effect of Ascorbate on the activity of hypoxia-inducible factors in cancer cells. Cancer Res..

[28] Grano A., De-Tullio M. (2007). Ascorbic acid as a sensor of oxidative stress and a regulator of gene expression: The yin and yang of Vitamin C. Med. Hypotheses.

[29] Rezvani R.H., Ali N., Nissen L.J., Harfouche G., Verneuil H.D. (2011). HIF-1α in epidermis: Oxygen sensing, cutaneous angiogenesis, cancer, and non-cancer disorders. J. Investig. Dermatol..

[30] Galoforo S., Berns C., Erdos G., Corry P., Lee Y. (1996). Hypoglycemia induced AP-1 transcription factor and basic fibroblast growth factor gene expression in multidrug resistant human breast carcinoma MCF-7/ADR cells. Mol. Cell. Biochem..

[31] Riester M., Xu Q., Moreira A., Zheng J., Michor F., Downey R.J. (2018). The warburg effect: Persistence of stem-cellmetabolism in cancers as a failure of differentiation Annalsof. Oncology.

[32] Spitz D., Sim J., Ridnour L., Galoforo S., Lee Y. (2000). Glucose deprivation-induced oxidative stress in human tumor cells: A fundamental defect in metabolism?. Ann. N. Y. Acad. Sci..

[33] de Groot S., Pijl H., van der Hoeven J.J.M., Kroep J.R. (2019). Effects of short-term fasting on cancer treatment. J. Exp. Clin. Cancer Res..

[34] Abdelwahab M., Fenton K., Preul M., Rho J., Lynch A., Stafford P., Scheck A. (2012). The ketogenic diet is an effective adjuvant to radiation therapy for the treatment of malignant glioma. PLoS ONE.

[35] Caccialanza R., Cereda E., De-Lorenzo F., Farina G., Pedrazzoli P. (2018). To fast, or not to fast before chemotherapy, that is the question. BMC Cancer.

[36] Zhao F., Keating A. (2007). Functional properties and genomics of glucose transporters. Curr. Genom..

[37] Macheda M., Rogers S., Best J. (2005). Molecular and cellular regulation of glucose transporter (GLUT) proteins in cancer. J. Cell. Physiol..

[38] Younes M., Brown R., Stephenson M., Gondo M., Cagle P. (1997). Overexpression of Glut1 and Glut3 in stage I non-small cell lung carcinoma is associated with poor survival. Cancer Cell.

[39] Rastogi S., Banerjee S., Chellappan S., Simon G. (2007). Glut-1 antibodies induce growth arrest and apoptosis in human cancer cell lines. Cancer Lett..

[40] Chan K., Chan J., Chung K., Fung K. (2004). Inhibition of cell proliferation in human breast tumor cells by antisense oligonucleotides against facilitative glucose transporter 5. J. Cell. Biochem..

[41] Chen C., Li X., Zhang L., Min J., Chan J., Fung K., Wang S., Zhang L. (2002). Synthesis of antisense oligonucleotide-peptide conjugate targeting to GLUT-1 in HepG-2 and MCF-7 Cells. Bioconjug. Chem..

[42] Chan J., Kong S., Choy Y., Lee C., Fung K. (1999). Inhibition of glucose transporter gene expression by antisense nucleic acids in HL-60 leukemia cells. Life Sci..

[43] Granchi C., Fortunato S., Minutolo F. (2016). Anticancer agents interacting with membrane glucose transporters. MedChemComm.

[44] Chen Q., Espey M., Krishna M., Mitchell J., Corpe C., Buettner G., Shacter E., Levine M. (2005). Pharmacologic ascorbic acid concentrations selectively kill cancer cells: Action as a pro-drug to deliver hydrogen peroxide to tissues. Proc. Natl. Acad. Sci. USA.

[45] Du J., Martin S., Levine M., Wagner B., Buettner G., Wang S., Taghiyev A., Du C., Knudson C., Cullen J. (2010). Mechanisms of ascorbate-induced cytotoxicity in pancreatic cancer. Clin. Cancer Res..

[46] Du J., Cullen J., Buettner G. (2012). Ascorbic acid: Chemistry, biology, and the treatment of cancer. Biochim. Biophys. Acta.

[47] Takemura Y., Satoh M., Satoh K., Hamada H., Sekido Y., Kubota S. (2010). High dose of ascorbic acid induces cell death in mesothelioma cells. Biochem. Biophys. Res. Commun..

[48] Chen Q., Espey M., Sun A., Pooput C., Kirk K., Krishna M., Khosh D., Drisko J., Levine M. (2008). Pharmacologic doses of ascorbate act as a pro-oxidant and decrease growth of aggressive tumor xenografts in mice. Proc. Natl. Acad. Sci. USA.

[49] Lv H., Wang C., Fang T., Li T., Lv G., Han Q., Yang W., Wang H. (2018). Vitamin C preferentially kills cancer stem cells in hepatocellular carcinoma via SVCT-2. NPJ Precis. Oncol..

[50] Chen P., Yu J., Chalmers B., Drisko J., Yang J., Li B., Chen Q. (2012). Pharmacological ascorbate induces cytotoxicity in prostate cancer cells through ATP depletion and induction of autophagy. Anticancer Drugs.

[51] Gao P., Zhang H., Dinavahi R., Li F., Xiang Y., Raman V., Bhujwalla Z., Felsher D., Cheng L., Pevsner J. (2007). HIF-dependent antitumorigenic effect of antioxidants in vivo. Cancer Cell.

[52] Mikirova N., Casciari J., Rogers A., Taylor P. (2012). Effect of high-dose intravenous vitamin C on inflammation in cancer patients. J. Transl. Med..

[53] Kim H.N., Kim H., Kong J.M., Bae S., Kim Y.S., Lee N., Joo B., Lee S.K., Kim H.-R., Hwang Y. (2011). Vitamin C down-regulates VEGF production in B16F10 murine melanoma cells via p42/44 MAPK activation suppression. J. Cell. Biochem..

[54] Pathi S., Lei P., Sreevalsan S., Chadalapaka G., Jutooru I., Safe S. (2011). Pharmacologic doses of ascorbic acid repress specificity protein (Sp) transcription factors and Sp-regulated genes in colon cancer cells. Nutr. Cancer.

[55] Hardaway C., Badisa R., Soliman K. (2012). Effect of ascorbic acid and hydrogen peroxide on mouse neuroblastoma cells. Mol. Med. Rep..

[56] Klingelhoeffer C., Kämmerer U., Koospal M., Mühling B., Schneider M., Kapp M., Kübler A., Germer C., Otto C. (2012). Natural resistance to ascorbic acid induced oxidative stress is mainly mediated by catalase activity in human cancer cells, and catalase-silencing sensitizes to oxidative stress. BMC Complement. Altern. Med..

[57] Espey M., Chen P., Chalmers B., Drisko J., Sun A.Y., Levine M., Chen Q. (2011). Pharmacologic Ascorbate synergizes with gemcitabine in preclinical models of pancreatic cancer. Free Radic. Biol. Med..

[58] Martinotti S., Ranzato E., Burlando B. (2011). In vitro screening of synergistic ascorbate-drug combinations for the treatment of malignant mesothelioma. Toxicol. Vitr..

[59] Ong P., Chan S., Ho P. (2011). Differential augmentative effects of buthionine sulfoximine and ascorbic acid in As_2_O_3_-induced ovarian cancer cell death: Oxidative stress-independent and -dependent cytotoxic potentiation. J. Oncol..

[60] Herst P., Broadley K., Harper J., McConnell M. (2012). Pharmacological concentrations of ascorbate radio-sensitize glioblastoma multiforme primary cells by increasing oxidative DNA damage and inhibiting G2/M arrest. Free Radic. Biol. Med..

[61] Wei Y., Song J., Chen Q., Xing D. (2012). Enhancement of photodynamic antitumor effect with pro-oxidant ascorbate. Lasers Surg. Med..

[62] Fritz H., Flower G., Weeks L., Cooley K., Callachan M., McGowan J., Skidmore B., Kirchner L., Seely D. (2014). Intravenous Vitamin C and Cancer: A systematic review. Integr. Cancer Ther..

[63] Jacobs C., Hutton B., Ng T., Shorr R., Clemons M. (2015). Is there a role for oral or intravenous ascorbate (Vitamin C) in treating patients with cancer? A systematic review. Oncologist.

[64] Nauman G., Gray J.C., Parkinson R., Levine M., Paller C. (2018). Systematic review of intravenous ascorbate in cancer clinical trials. Antioxidants.

[65] Van Gorkom G.N.Y., Lookermans E.L., Van Essen C.H., Bos G.M.J. (2019). The effect of Vitamin C (ascorbic acid) in the treatment of patients with cancer: A systematic review. Plasma Nutr..

[66] Welsh J.L., Wagner B.A., van’t Erve T.J., Zehr P.S., Berg D.J., Halfdanarson T.R., Yee N.S., Bodeker K.L., Du J., Roberts J.L. (2013). Pharmacological ascorbate with gemcitabine for the control of metastatic and node-positive pancreatic cancer (PACMAN): Results from a phase I clinical trial. Cancer Chemother. Pharmacol..

[67] Bazzan A.J., Zabrecky G., Wintering N., Newberg A.B., Monti D.A. (2018). Retrospective evaluation of clinical experience with intravenous ascorbic acid in patients with cancer. Integr. Cancer Ther..

[68] High-Dose Vitamin C (PDQ^®^)–Health Professional Version—National Cancer Institute. https://www.cancer.gov/about-cancer/treatment/cam/hp/vitamin-c-pdq.

[69] Cameron E., Campbel A. (1991). Innovation vs. quality control: An ‘unpublishable’ clinical trial of supplemental ascorbate in incurable cancer. Med. Hypotheses.

[70] Murata A., Morishige F., Yamaguchi H. (1982). Prolongation of survival times of terminal cancer patients by administration of large doses of ascorbate. Int. J. Vitam. Nutr. Res..

[71] Ma Y., Chapman J., Levine M., Polireddy K., Drisko J., Chen Q. (2014). High-dose parenteral Ascorbate enhanced chemosensitivity of ovarian cancer and reduced toxicity of chemotherapy. Sci. Transl. Med..

[72] Hoffer L.J., Robitaille L., Zakarian R., Melnychuk D., Kavan P., Agulnik J. (2015). High-dose intravenous Vitamin C combined with cytotoxic chemotherapy in patients with advanced cancer: A phase I-II clinical trial. PLoS ONE.

[73] Gunes-Bayir A., Kiziltan H.S. (2015). Palliative Vitamin C application in patients with radiotherapy-resistant bone metastases: A retrospective study. Nutr. Cancer.

[74] Zhao H., Zhu H., Huang J., Zhu Y., Hong M., Zhu H., Zang J., Li S., Yang L., Lian Y. (2018). The synergy of Vitamin C with decitabine activates TET2 in leukemic cells and significantly improves overall survival in elderly patients with acute myeloid leukemia. Leuk. Res..

[75] Wang F., He M.M., Wang Z.X., Li S., Jin Y., Ren C., Shi S.M., Bi B.T., Chen S.Z., Lv Z.D. (2019). Phase I study of high-dose ascorbic acid with mFOLFOX6 or FOLFIRI in patients with metastatic colorectal cancer or gastric cancer. BMC Cancer.

[76] Monti D.A., Mitchell E., Bazzan A.J., Littman S., Zabrecky G., Yeo C.J., Pillai M.V., Newberg A.B., Deshmukh S., Levine M. (2012). Phase I evaluation of intravenous ascorbic acid in combination with gemcitabine and erlotinib in patients with metastatic pancreatic cancer. PLoS ONE.

[77] Polireddy K., Dong R., Reed G., Yu J., Chen P., Williamson S., Violet P.-C., Pesetto Z., Godwin A.K., Fan F. (2017). High dose parenteral ascorbate inhibited pancreatic cancer growth and metastasis: Mechanisms and a phase, I/II a study. Sci. Rep..

[78] Raymond Y., Glenda C., Meng L. (2016). Effects of high doses of Vitamin C on cancer patients in singapore: Nine cases. Integr. Cancer Ther..

[79] Schoenfeld J.D., Sibenaller Z.A., Mapuskar K.A., Wagner B.A., Cramer-Morales K.L., Furqan M., Sandhu S., Carlisle T.L., Smith M.C., Hejleh T.A. (2017). O_2_- and H_2_O_2_-mediated disruption of Fe metabolism causes the differential susceptibility of NSCLC and GBM cancer cells to pharmacological ascorbate. Cancer Cell.

[80] Nielsen T.K., Hojgaard M., Andersen J.T., Jorgensen N.R., Zerahn B., Kristensen B., Henriksen T., Lykkesfeldt J., Mikines K.J., Poulsen H.E. (2017). Weekly ascorbic acid infusion in castration-resistant prostate cancer patients: A single-arm phase II trial. Transl. Androl. Urol..

[81] Hoffer L.J., Levine M., Assouline S., Melnychuk D., Padayatty S.J., Rosadiuk K. (2008). Phase I clinical trial of i.v. ascorbic acid in advanced malignancy. Ann. Oncol..

[82] Riordan H.D., Casciari J.J., Gonzalez M.J., Riordan N.H., Miranda-Massari J.R., Taylor P., Jackson J.A. (2005). A pilot clinical study of continuous intravenous ascorbate in terminal cancer patients. Puerto Rico Health Sci. J..

[83] Stephenson C.M., Levin R.D., Thomas Spector T., Lis C.G. (2013). Phase I clinical trial to evaluate the safety, tolerability, and pharmacokinetics of high-dose intravenous ascorbic acid in patients with advanced cancer. Cancer Chemother. Pharmacol..

[84] Buettner G.R. (1988). In the absence of catalytic metals ascorbate does not autoxidize at pH 7: Ascorbate as a test for catalytic metals. J. Biochem. Biophys. Methods.

[85] Padayatty S.J., Levine M. (2016). Vitamin C physiology: The known and the unknown and Goldilocks. Oral Dis..

[86] Korth H., Meier A., Auferkamp O., Sicking W., de-Groot H., Sustmann R., Kirsch M. (2012). Ascorbic acid reduction of compound I of mammalian catalases proceeds via specific binding to the NADPH binding pocket. Biochemistry.

[87] Englard S., Seifter S. (1986). The biochemical functions of ascorbic acid. Annu. Rev. Nutr..

[88] Levine M. (1986). New concepts in the biology and biochemistry of ascorbic acid. N. Engl. J. Med..

[89] Levine M., Padayatty S.J., Wang Y., Corpe C., Lee L., Wang J., Chen Q., Zhang L., Stipanuk M., Caudill M. (2006). Vitamin C. Biochemical, Physiological, and Molecular Aspects of Human Nutrition.

[90] Chen Q., Espey M.G., Sun A.Y., Lee J.-H., Krishna M.C., Shacter E., Choyke P.L., Pooput C., Kirk K.L., Buettner G.R. (2007). Ascorbate in pharmacologic concentrations selectively generates ascorbate radical and hydrogen peroxide in extracellular fluid in vivo. Proc. Natl. Acad. Sci. USA.

[91] Liu M., Ohtani H., Zhou W., Ørskov A.D., Charlet J., Zhang Y.W., Shen H., Baylin S.B., Liang G., Grønbæk K. (2016). Vitamin C increases viral mimicry induced by 5-aza-20 -deoxycytidine. Proc. Natl. Acad. Sci. USA.

[92] Linowiecka K., Foksinski M., Brozyna A.A. (2020). Vitamin C Transporters and Their Implications in Carcinogenesis. Nutrients.

[93] Carosio R., Zuccari G., Orienti I., Mangraviti S., Montaldo P.G. (2007). Sodium Ascorbate induces apoptosis in neuroblastoma cell lines by interfering with iron uptake. Mol. Cancer.

[94] Lynn M. (1970). Origin of Eukaryotic Cells.

[95] Fenton H.J.H. (1894). Oxidation of Tartaric in presence of Iron. J. Chem. Soc. Trans..

[96] Sanchez M., Sabio L., Galvez N., Capdevila M., Dominguez-Vera J.M. (2017). Iron Chemistry at the Service of Life. Int. Union Biochem. Mol. Biol..

[97] Johnson D.C., Dean D.R., Smith A.D., Johnson K. (2005). Structure, function, and formation of biological iron-sulfur clusters. Annu. Rev. Biochem..

[98] Guo Y., Echavarri-Erasun C., Demuez M., Jiménez-Vicente E., Bominaar E.L., Rubio L.M. (2016). The Nitrogenase FeMo-Cofactor Precursor Formed by NifB Protein: A Diamagnetic Cluster Containing Eight Iron Atoms. Angew. Chem. Int. Ed. Engl..

[99] Stokkermans J.P.W., Pierik A.J., Wolbert R.B.G., Hagen W.R., Van Dongen W.M.A.M., Veeger C. (1992). The primary structure of protein containing a putative [6Fe-6S] prismane cluster from desulfovibrio vulgaris (Hildenborough). Eur. J. Biochem..

