# Supplementary material for: The Result of Vitamin C Treatment of Patients with Cancer: Conditions Influencing the Effectiveness
Source: Int J Mol Sci. 2022 Apr 15;23(8):4380. doi: 10.3390/ijms23084380 (PMC9030840; doi:10.3390/ijms23084380)
Supplement: Supplementary file 1 [file ijms-23-04380-s001.zip › ijms-1540237-supplementary.pdf]

International Journal of
Molecular Sciences

MDPI

Supplmentary Materials

The Hypothesis of the Structures for Energy Transformation in Living Cells Vitamin C, the Spark Plug of Glycolysis

János Hunyady

Department of Dermatology, Medical Faculty, University of Debrecen, 4032 Debrecen, Hungary; hunyadi@med.unideb.hu

1. Structures for Energy Transformation

Unique Structures for Energy Transformation (SETs) are responsible for producing energy and ATP and maintaining the membrane potential by H^+ production. The different SETs contain individual building blocks, resulting in different efficiencies. Eukaryotic cells utilize the SET of Aerobic Glycolysis (SET-AG) and the SET of Oxidative Phosphorylation (SET-OP), allowing them to live in an oxygen-rich and anoxic environment. The scientific explanation underlining this hypothesis is described in the publication titled: The Role of Vitamin C in the Energy Supply of Cells Hypothetical Structure for Energy Transformation [8].

The adenosine diphosphate-producing unit (ADP-PU) and Complex V are the defining components of SET-AG and SET-OP. The ADP-PU is built by structural rudiments forming a nest and waiting for parent molecules to be transformed. Unique molecules help the movement and the positioning of the parent molecules.

Fe-S clusters (probably two [6Fe-6S]), one nicotinamide, one flavine molecules are the essential structural elements, and four NH_3, four uric acids, two L- ascorbic acids (AA), six D-glucose, and twelve $H_2PO_4^-$ molecules are the parent molecules of the ADP-PU (Table S1). SET- AG is built by three ADP -PUs, producing 3 x (four ATP, two Pyruvate originated lactate, ten CO_2), and energy. The SET-OP consists of 3 SET-AG, a Pyruvate dehydrogenase complex, and three high molecular weight cytochromes, producing 3 × 3 x (four ATP, sixteen CO_2) and energy.

2. Three Working-Phases of ADP Producing Unit and Complex V

ADP-PUs and Complex V are working in three-phase. The first phase is the loose phase when molecules arrive in the unit. The second active phase is when the products are prepared. Finally, during the third open phase, the molecules leave the Unit (Figure S1.)

Table S1. Determining Molecules of Structures for Energy Transformation in the ADP producing Unit of SET-AG and SET-OP.

Structure Molecules		
[Fe-S] cluster<br>Two [Fe-S] clusters offering 2x12 oxygen binding places.(probably two [6Fe-6S)	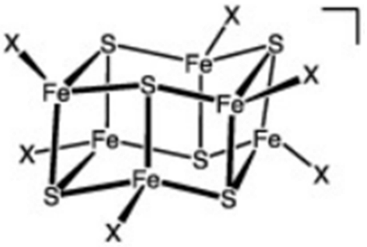<br><br>[6Fe-6S]	2

Flavin	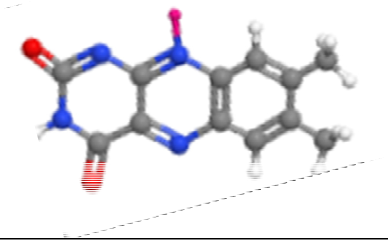	1
Nicotinamide	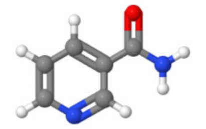	1
**Parents molecules**		
Uric acid→Adenine	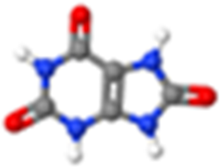 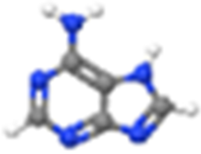	4
$H_2PO_4^-$	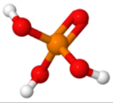	12
L-ascorbic acid	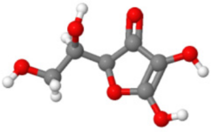	2
D-glucose	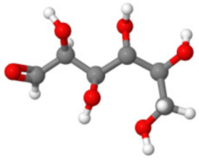	6
NH_3	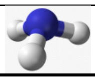	4

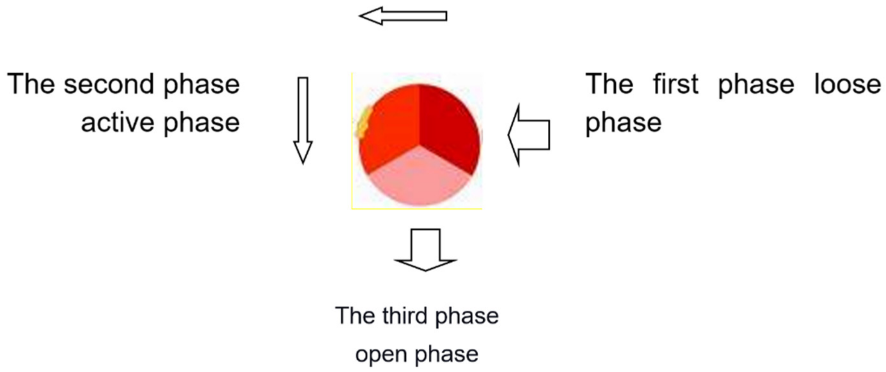

Figure S1. The three phases of ADP-PU and Complex V.

ADP-PU and Complex V's synchronized function ensures the constant electron transfer, the maintenance of membrane potential, and the continuous ATP synthesis https://set.suicidevolution.com/v2/ (accessed on 27 June2021).

3. Vitamin C, Dehydroascorbic Acid.

Vitamin C is an essential nutrient involved in tissue repair and collagen formation. It is a co-factor for enzymes and a co-substrate. AA is an electron donor and, therefore, a reducing agent. Despite its ability to act as a reducing agent, ascorbate may also play an essential role as an oxidizing factor. The oxidation process of ascorbate mainly occurs in catalytic metals [84]. AA is involved in various body functions due to its unique properties in redox homeostasis [85]. Vitamin C oxidizes rapidly and converts to dehydroascorbic acid (DHA) upon a pH change. DHA and ascorbate radicals can also be reversibly reduced to ascorbate [46].

4. Vitamin C is the Sustaining Molecule in the Energy Conversion Process

Korth et al., published that vitamin C molecules are in the pocket of NADPH, presumably at the adenine binding site of the mitochondrial inner membrane [86]. Partly supported by this observation, we created our hypothesis regarding the SETs. Two [Fe-S] clusters are the defining element of ADP-PU. The two AAs of the Unit enable the Fe^{3+} to Fe^{2+} conversion, initiating the process that results in ADP, HPO_3^{2e-} and aminated uric acid production.

AA functions as an electron donor for fifteen mammalian and three fungal enzymes [87–89]. The effect of vitamin C oxidation is the formation of hydrogen peroxide, which can influence cellular metabolism by altering intracellular redox stability [84,90].

In addition, vitamin C can act as a pro-drug due to its ability to work as a reducing and oxidizing factor [90,91]. Therefore, the concentration of vitamin C in plasma determines the preferred form of vitamin C. At physiological concentrations, AA preferentially exerts its antioxidant functions. On the other hand, higher AA levels are associated with pro-oxidant functions [90]. However, the oxidation process of ascorbate mainly occurs in the presence of catalytic metals.

Vitamin C plays an essential role in restoring the activity of 2-oxoglutarate and Fe^{2+} -dependent dioxygenases (2-OGDD). Ascorbic acid crucially increases the rate of the reaction catalyzed by 2-OGGD by targeting its catalytic domain and regenerating iron ions from Fe^{3+} to Fe^{2+} [92]. Based on this publication, we suppose that a continuous AA- dehydro-AA - AA conversion might cause a change of the Fe ions (Fe^{3+} - Fe^{2+} - Fe^{3+}) in the Fe-S clusters of ADP-PUs, resulting in constant energy and ATP production and the permanent maintenance of the membrane potential.

A high dose of ascorbic acid results in cancer cell death in defined circumstances [46,90]. The specific mechanism of vitamin C-induced apoptosis is due to a perturbation of intracellular iron levels. In the absence of catalytic metals, ascorbate does not autoxidize [84]. Furthermore, in vitro experiments show that apoptotic markers were reverted in a dose- and time-dependent manner when the cells were pretreated with the iron donor ferric ammonium citrate [93]. Therefore, we predict that Fe-S clusters are critical inductors of the toxic reaction.

5. The Two-Fold Energy Supply of The Eukaryotic Cells

The first eukaryotic cells arose from the symbiosis of an ancient cell (originally living in an O_2-free environment) with an O_2 using cell, now known as the mitochondrion [94]. The ancient cell uses SET-AG in the cytoplasm and the glycosomes or peroxysomes, while mitochondria use SET-OP. Correspondingly, eukaryote cells possess SET-AG and SET-OP. Thus, they can live in an oxygen-rich and anoxic environment [8].

6. Iron Chemistry at the Service of Life

Iron is necessary for several crucial processes such as hemoglobin and myoglobin transport, and oxygen storage in mammals. Thus it is a vital element for almost all organisms on Earth. In addition, iron supports electron transfer in various iron-sulfur protein or cytochrome reactions. Living organisms take up iron as the primary metal to carry out

all of these functions due to its abundance in the Earth's crust and oceans and because of the rich coordination chemistry of its two primary redox states, Fe^{2+} and Fe^{3+}.

Iron chemistry within biology is an example of how organisms evolved by creating molecular machinery to perform crucial processes with extraordinary elegance and efficiency.

6.1. Fenton Reaction

It has been well established that free Fe^{2+} promotes ROS formation via distinct pathways like the Fenton reaction [95]. In 1894, Fenton described the strongly oxidizing properties of a mixture of Fe^{2+} and H_2O_2. Haber & Weiss proved that the hydroxyl radical OH^{e-} is an active species formed by one-electron transfer from Fe^{2+} to H_2O_2 [96]. The formation of OH^{e-} radicals usually refers to the presence of free Fe^{2+} traces of unprotected Fe^{3+} existing in many biological systems can also be sufficient to generate OH^{e-} radicals. The following one-electron reduction reaction forms Fe3+ + O^{2e-} Fe^{2+} + O_2. Living organisms can avoid Fenton reactions by controlling the Fe^{3+}/Fe^{2+} redox potential with the coordination of some biomolecules.

6.2. Fe-S Clusters

The combination of the chemical reactivity of iron and sulfur and the many variations in cluster composition, oxidation states, and protein environments, allow Fe-S clusters to participate in numerous biological processes. Fe-S clusters are essential for redox catalysis in nitrogen fixation, mitochondrial respiration, photosynthesis, regulation of vital metabolic pathways (i.e., cellular iron homeostasis and oxidative stress response), and nuclear genome replication and maintenance. The most abundant Fe-S clusters are of the rhombic [2Fe-2S] and cubic [4Fe-4S] types, but [3Fe-4S] [4Fe-3S], [6Fe-6S] and [8Fe-8S] clusters have also been described [97–99]. The property of iron (Fe^{2+}; Fe^{3+}) and its affinity for binding to sulfur or oxygen determine the possessions of the SET's Fe-S clusters. The binding relationship of sulfur to Fe^{2+} is higher than that of oxygen. Accordingly, the binding of sulfur and oxygen to iron is determined by the nature of Fe. In the case of Fe^{2+}, the binding of sulfur; in the case of $Fe^{3+,}$ the binding of oxygen is realized.

7. Hypothesis

7.1. The Adenosine Diphosphate Producing Unit of SET

The adenosine diphosphate-producing unit (ADP-PU) is the determining component of SET-AG and SET-OP. ADP-PU and Complex V work synchronously to produce ATP; https://set.suicidevolution.com/v2/.

SET-ADP-PUs are responsible for making ADP. The structural rudiments form a nest and wait for the parent molecules in ADP-PU, where the parent molecules are converted.

The ADP-PUs of SET-AG and SET-OP have two [Fe-S] clusters with 2 × 12 oxygen-binding places, one Flavin, one nicotinamide molecule. The parent molecules are four NH_3, four uric acids (four AU-originated adenine), two L- AA, and six D-glucose (Figure S2).

One Complex V + three ADP-PU form the SET-AG, producing 3 x (four ATP, two Pyruvate originated lactate, ten CO_2, 8 H^+), and energy (Figure 2). The SET-OP consists of 3 SET-AG, Pyruvate dehydrogenase complex, and high molecular weight cytochromes producing 3 × 3 x(4 ATP+ 16 CO_2 + 8 H^+) + energy [8].

ADP-Producing Unit: Two [Fe-S] clusters + nicotinamide + flavine; + 4 aminated UA + 4 UA + 4 NH_3 + 12 $H_2PO_4^-$ + 6 D-glucose + two L-ascorbic acid = 4 ADP + 4 HPO_3^{2e-} + 4 Pyruvate (lactate) + two new L-ascorbic acid + 4 aminated UA molecules.

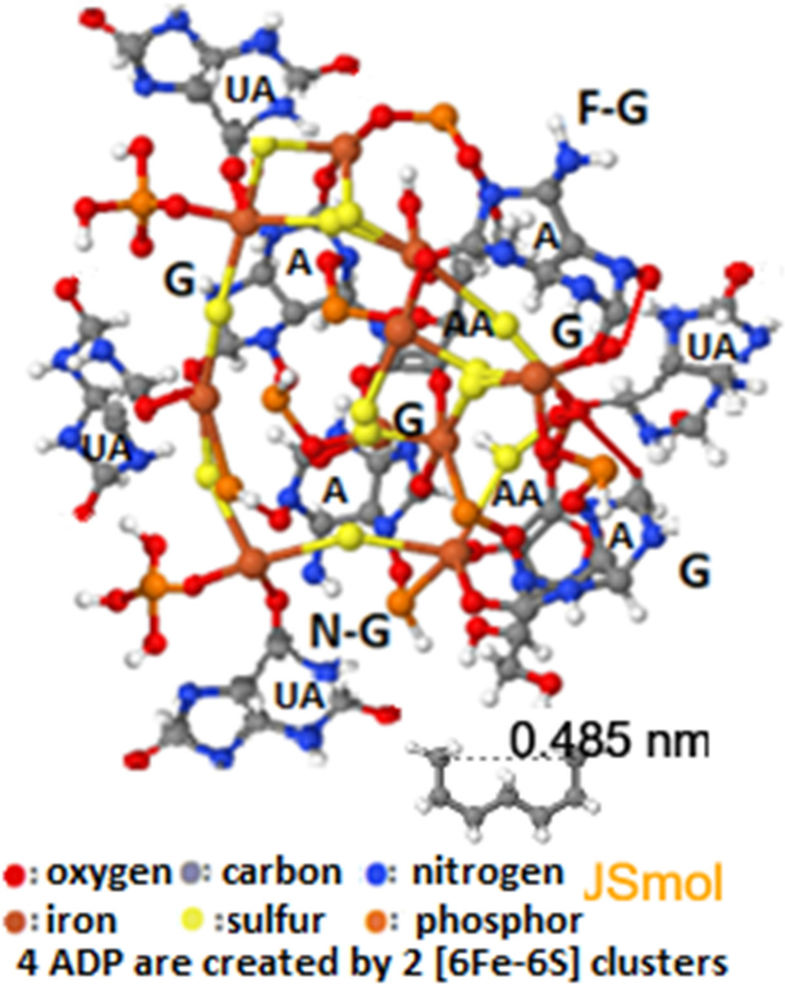

Figure S2. The molecular composition of the ADP-Producing Unit (montage of several images). Two [Fe-S] clusters, one Flavin, one nicotinamide, four UA, four UA originated adenine, two L-AA, and six D-glucose molecules. UA: Uric Acid; F-G: Flavine-D-glucose (not shown); G: D-glucose (not shown); A: Adenine; AA: L-Ascorbic Acid; N-G: Nicotinamide-D-glucose (not shown) (https://set.suicidevolution.com/v2/).

8. Energy Conversion in the SETs

8.1. Vitamin C, the Spark Plug of Glycolysis

Based on the publication by Linowiecka et al. [87], we hypothesize that AA is the spark plug of glycolysis. Therefore, we assume that a continuous AA - dehydro-AA - AA conversion might cause a permanent alternation of Fe ions (Fe^{3+} - Fe^{2+} - Fe^{3+}) in the Fe-S clusters of ADP -PUs leading to constant energy and ATP production and maintenance of membrane potential.

Two Fe^{3+} + AA = two Fe^{2+} + DHA + $2H^+$

Two Fe^{2+} + DHA + two $H_2PO_4^-$ = two Fe^{3+} + AA + two HPO_3^{2e-} + two O^{2e-}

Two [Fe-S] clusters are the defining structures of all ADP -PUs.

The Fe-S clusters produce 24 O^{2e-}, creating ten CO_2 and four H_2O molecules.

The Fe-S clusters are built up by [2Fe-2S] units. There are two forms of the [2Fe-2S] units: [2Fe-2S]a is responsible for the amination of UA and HPO_3^{2e-} production, while [2Fe-2S]b is producing only HPO_3^{2e-}.

Each [Fe-S] clusters are formed by one [2Fe-2S]a + two [2Fe-2S]b.

8.2. The Function of the [2Fe-2S]a Unit, Formations of Aminated Uric Acid, HPO_3^{2e-}, H_2O, And CO_2

In the [2Fe-2S]a unit, two uric acids, two $H_2PO_4^{e-}$, and two NH_3 molecules met the Fe-S cluster. First, AA converts Fe^{3+} ions to Fe^{2+} while DHA and two H^+ are formed. Then, two oxygen atoms from two UA (the oxygen atom C6) and two oxygen atoms from two $H_2PO_4^{e-}$ molecules bond to the Fe-S cluster (A). Then, a DHA converts the Fe^{2+} to Fe^{3+}, resulting in the exchange of sulfur atoms with oxygen forming two $NH\text{-}UA^{e-}$, two HPO_3^{2e-}, and four O^{e2-} (B). Subsequently, two UA, two $H_2PO_4^{e-}$ and two NH_3 arrive into the cluster (C) (Figure S3).

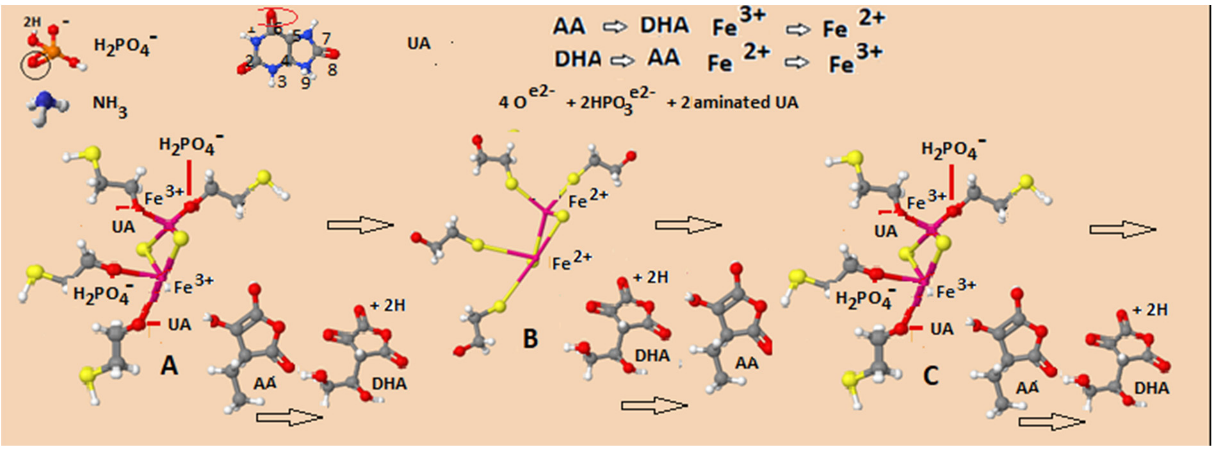

Figure S3. Production of aminated uric acid, HPO_3^{2e-}, H_2O, and CO_2 by the [2Fe-2S]a cluster. Abbreviations: UA: uric acid; AA: ascorbic acid; DHA: dehydroascorbic acid.

8.3. The Function of the [2Fe-2S]b Unit, Formations of HPO_3^{2e-}, and CO_2

In the [2Fe-2S]b unit, two aminated uric acid and two $H_2PO_4^{e-}$ molecules met the Fe-S cluster. First, AA converts Fe^{3+} ions to Fe^{2+} while DHA and two hydrogen atoms are formed. As a result, four O^{2e-} ions and two HPO_3^{e2-} leave the cluster (A, B). after that, a DHA converts the Fe^{2+} to Fe^{3+}. Subsequently, two $H_2PO_4^{e-}$ and two aminated uric acid molecules (the oxygen atom C2 and the oxygen atom of C7; circled) are bound to the Fe-S cluster (C) (Figure S4).

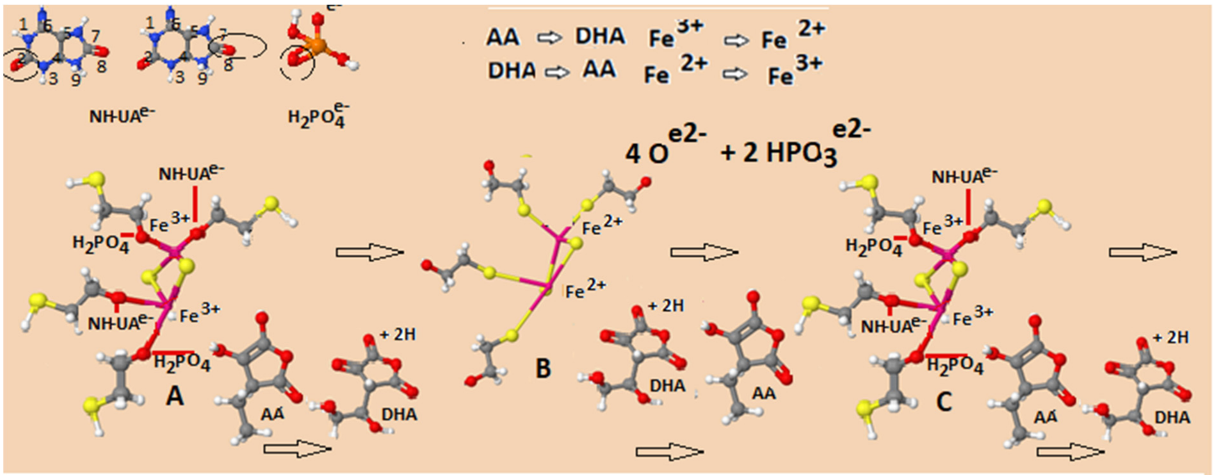

Figure S4. Production of HPO_3^{2e-} and CO_2 by [2Fe-2S]b cluster. Abbreviations: UA: uric acid; AA: ascorbic acid; DHA: dehydroascorbic acid.

8.4. Structures for Aerobe-Glycolysis and Oxidative Phosphorylation

ADP-PU is supplemented with diverse Fe-S clusters in cells with different properties. The characteristics of these clusters determine the nature and the products of the structure.

SET-AG consists of 3 ADP-PUs, each characterized by two [Fe-S] clusters, each obtaining two [2Fe-2S]a + four [2Fe-2S]b clusters.

SET-OP consists of 9 ADP-PUs, each characterized by two [Fe-S] clusters, each obtaining two [2Fe-2S]a + four [2Fe-2S]b clusters Table S2).

8.5. SET of Aerobe Glycolysis

Two [Fe-S] clusters are the determinant structures of SET-AG's ADP-PUs. One of the clusters serves the NAD part of the Unit, while the other supplies the FAD part. The transformation starts with the reaction of the two AA resulting in the change of the iron ion from Fe^{3+} to Fe^{2+}. As a result, oxygen-containing molecules (uric acid and $H_2PO_4^{e-}$) will arrive in the clusters. The two [Fe-S] clusters offer places for 24 oxygen atoms. They come from the eight uric acids (4 × 1+4 × 2) and twelve $H_2PO_4^{e-}$ molecules.

[Fe-S] cluster + nicotinamide + flavine, + 4UA+ $4NH_3$ + 12 $H_2PO_4^{e-}$ + 6 D-glucose + 2 AA = 4 ADP + 2 Pyruvate (lactate) + 2 New AA. In addition four aminated UAs are produced for the next turn of transformation and four HPO_3^{2e-} molecules used up for ATP synthesis in the Cytochrome V.

The structure of the ADP-PU's Fe-S clusters allows the electron transfer's realization. Figure S5 presents the bounding of two aminated UA and two $H_2PO_4^{e-}$ molecules to the Fe-S cluster. One hydrogen atom of the $H_2PO_4^{e-}$ molecule (arrows) might change the Fe^{2+} to Fe^{3+}, resulting in sulfur – oxygen change in the cluster. In addition, one dehydroascorbic acid might catalyze the reaction (Figure S5).

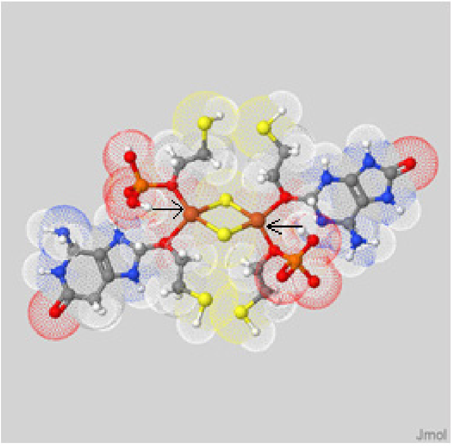

Figure S5. Two aminated uric acid molecules and two $H_2PO_4^{e-}$ molecules arrive in the Fe-S cluster. The hydrogen of the $H_2PO_4^{e-}$ molecule, with the help of dehydroascorbic acid, is supposed to change back the Fe^{2+} to Fe^{3+}.

The produced O^{2e-} atoms cause the oxidation of the 5th carbon atoms of the two AA molecules and the glucose – ribose transformation (Figure S6). Four ribose will form adenosine, while the other two will be split into pyruvate (forming lactate) and acetate. Set-AG's ADP-PU creates four ADP. The twenty-four O^{2e-} atoms make ten CO_2 and four H_2O molecules.

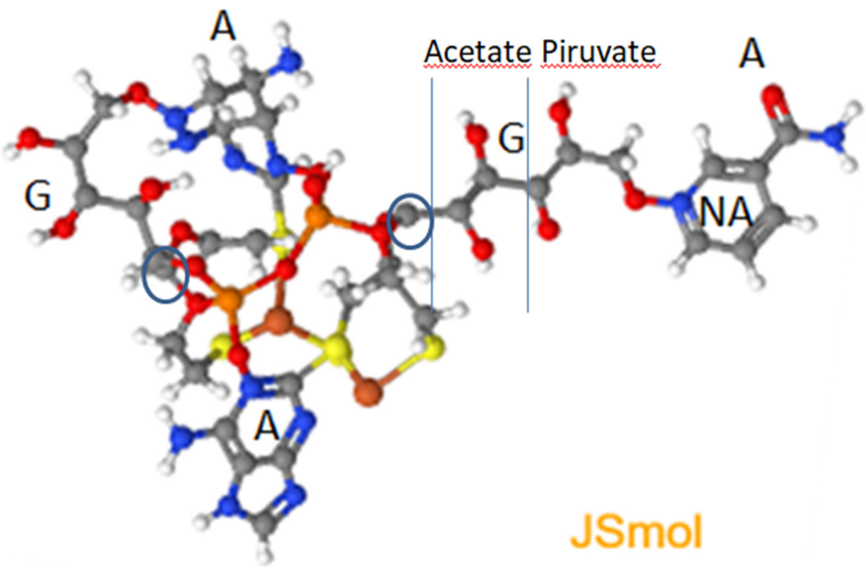

Figure S6. Glucose-ribose transformation. The 6th carbon atoms of two D-glucose molecules are oxidized (circled), resulting in the D-glucose – ribose transformation. G: D-glucose; A: Adenine¸NA: Nicotinamide.

9. The Hypothetical Development of Cellular Energy Supply

The hypothetical evolution of energy supply is summarized in Table S2.

Table S2. The supposed evolution of energy supply.

**Primitive Cell Membrane**	**Fe-S Clusters, UA, Nicotinamide, Flavine, $H_2PO_4^{e-}$, D-Glucose, L-AA**		
Primitive cell	Fe-S clusters, Complex V, SET ADP-PU		
O_2	No	No	yes
	Anaerob glycolysis	Aerob glycolysis	Oxidative phosphorylation
	SET-ANG	SET-AG	SET-OP
			3SET-AG+PDC
		Genom1	Genom2
Eucariots	SET-AG + SET-OP		
The development of a multi-colored complex living world with the Darwinian evolution			

UA: uric acid; L-AA: L-ascorbic acid; ADP-PU: ADP-Producing Unit; SET-ANG: Structure for Energy Transformation with ANaerobic Glycolysis; SET-AG: Structure for Energy Transformation with Aerobic Glycolysis; SET-OP: Structure for Energy Transformation with Oxidative Phosphorylation; PDC: Pyruvate Dehydrogenase Complex.

9.1. The Synchronized Function of the Three ADP-PUs and Complex V

The ADP-PU is the primary determining unit of all SETs. The SET-AG consists of three ADP-PUs. These units work synchronized with the Complex V. When the ADP-PU is in the open phase, releasing ADP and HPO_3^{2e-}, the Complex V is in the Enter phase, ready to accept them and make ATP (Table S3, Figures S7 and S8).

SET AG is located in the plasma membrane, in the glycosomes, and the peroxysomes of the cells.

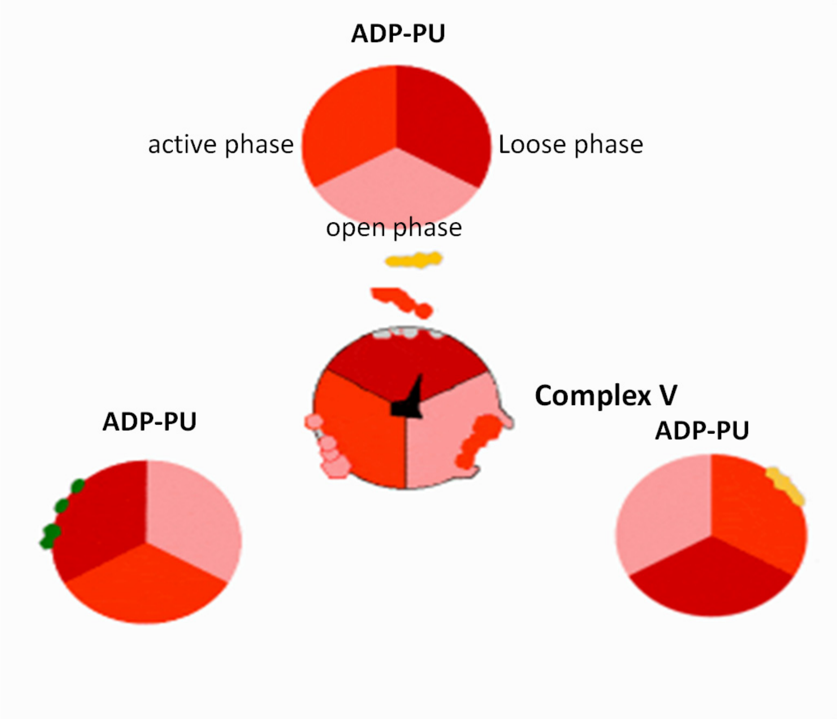

Figure S7. Cooperation of the three ADP-PU Structure with Complex V in the Energy Transformation Structure for Aerobic Glycolysis.

9.2. The Synchronized Function of the Three (3 ADP-PUs + Complex V

The Structure for AG contains nine ADP-Pus + three Complex V. The synchronized function of three times (3 ADP-PU+Complex V) (a,b,c) results in the continuous maintenance of the merman potential and the permanent ATP production (Figure S8).

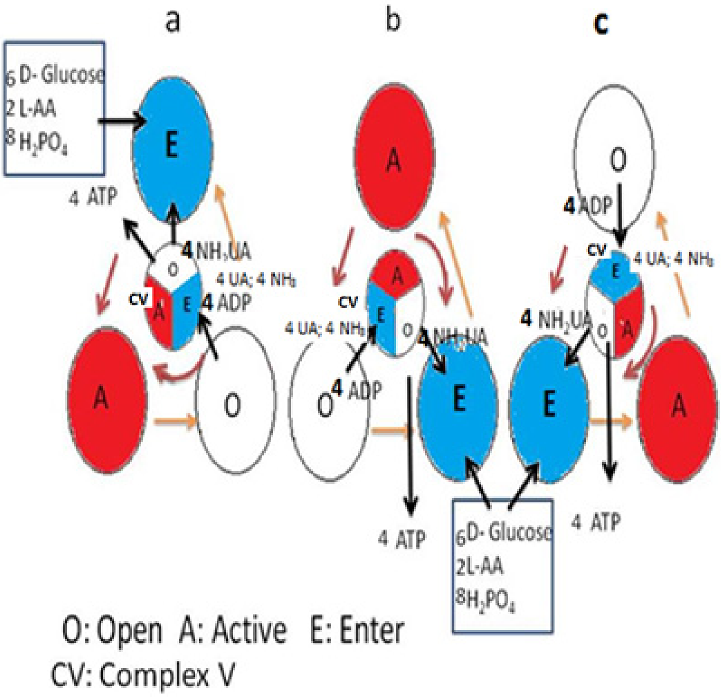

Figure S8. The supposed cooperation of the three ADP-Producing Units (**a**–**c**) and the Complex V of the Structures for Energy Transformation.

10. SET of Oxidative Phosphorylation

The SET-OP consists of three SET-AGs + three high molecular cytochromes + three Pyruvate Dehydrogenize Complex (PDC). Accordingly, the Pyruvate molecules are oxidized, resulting in more energy. SET OP is located in the mitochondrial crista.

10.1. Continuous Electron and ATP Production, the Two Steps of ATP Production

The process of energy transformation starts with [Fe-S] clusters. In the first step, aminated uric acid, HPO_3^{2e-} and ADP molecules are produced. Finally, Complex V will produce ATP from ADP + HPO_3^{2e-} Table S3.

Table S3. The process of ATP production.

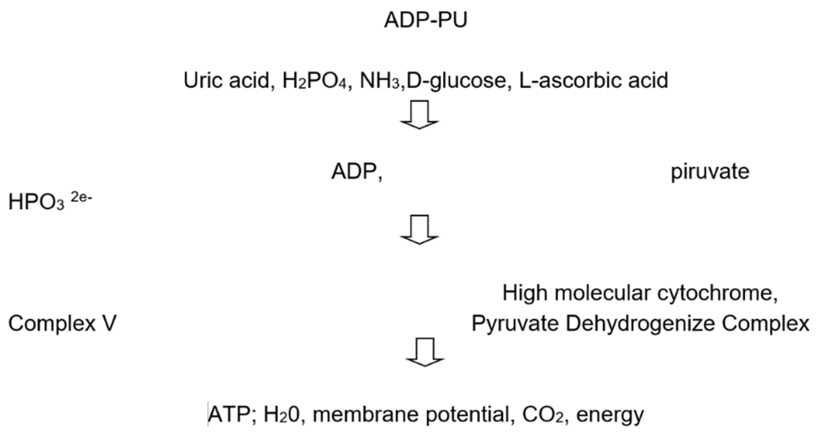

10.2. The Complete Structures for Energy Transformation

Each SET is assumed to be made up of 3 or 9 (3 × 3) ADP-PUs. In SET AG, three similar units are forming the SET. Each unit is complemented by Complex V. SET OP consisting of three SET-AGs, three Hmc, and three Pyruvate Dehydrogenize Complex.

10.3. Cancer and the Vitamin C

A high dose of Vitamin C is cytotoxic for cancer cells in defined circumstances because it starts the process of energy transformation. During the conversion, ribose is prepared from glucose. In the case of glucose deficiency, the produced O^{2e-} can kill the cell in defined situations. Primarily, tumor cells using SET-AG are sensitive to Vitamin C as mitochondria provide adequate protection against free radicals in the presence of O_2.
